# Factoid Question Answering with Distant Supervision

**DOI:** 10.3390/e20060439

**Published:** 2018-06-05

**Authors:** Hongzhi Zhang, Xiao Liang, Guangluan Xu, Kun Fu, Feng Li, Tinglei Huang

**Affiliations:** 1 Key Laboratory of Technology in Geo-spatial Information Processing and Application System, Institute of Electronics, Chinese Academy of Sciences, Beijing 100190, China; 2 School of Electronic, Electrical and Communication Engineering, University of Chinese Academy of Sciences, Beijing 100049, China; 3 Institute of Electronics, Chinese Academy of Sciences, Suzhou, Suzhou 215123, China

**Keywords:** distant supervision, question answering, reading comprehension, question paraphrase

## Abstract

Automatic question answering (QA), which can greatly facilitate the access to information, is an important task in artificial intelligence. Recent years have witnessed the development of QA methods based on deep learning. However, a great amount of data is needed to train deep neural networks, and it is laborious to annotate training data for factoid QA of new domains or languages. In this paper, a distantly supervised method is proposed to automatically generate QA pairs. Additional efforts are paid to let the generated questions reflect the query interests and expression styles of users by exploring the community QA. Specifically, the generated questions are selected according to the estimated probabilities they are asked. Diverse paraphrases of questions are mined from community QA data, considering that the model trained on monotonous synthetic questions is very sensitive to variants of question expressions. Experimental results show that the model solely trained on generated data via the distant supervision and mined paraphrases could answer real-world questions with the accuracy of 49.34%. When limited annotated training data is available, significant improvements could be achieved by incorporating the generated data. An improvement of 1.35 absolute points is still observed on WebQA, a dataset with large-scale annotated training samples.

## 1. Introduction

Question answering (QA) can facilitate the information acquisition from multiple data sources, such as structured knowledge bases (KB) [1,2], semi-structured tables [3] and free texts [4,5,6]. Automatic question answering is an important task for artificial intelligence community. Traditional QA systems, including the document based QA and KB based QA, are mainly built on information retrieval [7] and semantic parsing [8,9]. In recent years, QA methods based on deep learning are intensively studied along with the construction of large-scale QA datasets, such as SimpleQuestions [2], CNN Daily Mail [10] and Squad [4]. With the incorporation of effective mechanisms such as attention and contextual vectors [11], Wang et al. [12] has reported performance that is comparable to humans on the Squad dataset.

However, QA remains an unsolved problem [13] and further improvements are still needed for real-world applications. One of the drawbacks of deep learning methods is that lots of labeled data is needed to train the model with millions of parameters to avoid over-fitting. It is laborious and expensive to manually annotate large scale training data, thus hindering the application of deep learning models on QA of new domains or languages, where only limited annotated data is available. Motivated by this issue, we explore how to automatically generate training data for factoid QA. Notice that a typical QA system consists of a document retriever that retrieves the relevant document, and a QA model that extracts the answer from the given document. In this paper, we mainly focus on generating training data for the QA model.

There are some works [14,15] on question generation, where the QA models are enhanced utilizing their inter-correlation with question generation models. However, these methods are not applicable for the training of QA models with limited labeled data because their question generation models are also trained via supervised learning. By comparison, we study how to automatically generate QA pairs for the training of QA models by incorporating the knowledge base. The procedure of training data generation is illustrated in Table 1. The triple (Albert Einstein; birth place; Ulm, Kingdom of Württemberg, German Empire) from KB records the birth place of Albert Einstein. A simple question is synthesized using templates considering the subject and the predicate. The inherent relationship of triples ensures that the generated questions query the objects, so the objects could be regarded as the answers. Then, the distant supervision assumption is that a document having mentions of both the subject and the object within one sentence can be taken as evidence to answer the question, while the mention of the object can be tagged as the answer span.

There are billions of triples recorded in large-scale KBs such as Wikidata [16] and DBpedia [17], so great amounts of training data can be automatically generated.

Though a large number of QA pairs are generated, it is observed that the synthetic questions are different from the questions asked by people in both the content and the style:

- Content. The distribution of synthetic questions is inconsistent with that of the real-world questions. For example, users may seldom ask common sense questions, or questions whose answers could be inferred easily from entity names, such as gender of Madame Curie, or English name of Benjamin Franklin, while we observe that a certain proportion of triples record such information for the completeness of KB.
- Style. The sentence structures of the generated questions are monotonous. If trained on these data, the model will be very sensitive to various expressions of questions.

Twofold efforts are made to have the generated questions reflect the query interests and expression styles of users. On one hand, we propose selecting the generated questions by the probability that they are asked in the real world. Specifically, the probability is estimated by exploring community QA data to reflect the interests of users. The fact that a large portion of questions of community QA are about opinionated, experiential or controversial topics places a challenge on the estimation. On the other hand, preliminary effort is made to mine paraphrases of the synthetic questions. We search questions from community QA with the subject and the predicate as the keywords, and assume that retrieved questions with the object as the answer are the paraphrases of the synthetic question. We call this *distant paraphrase*.

Evaluations are performed on real-world questions. Up to 49.34% of the questions are correctly answered with the model solely trained on distant-supervised data, and distant paraphrase plays a vital role for the generalization of the model. We also investigate the situation where the model is firstly trained on the distant supervision data and then fine-tuned on the labeled data. Significant improvement is observed when the size of training data is small. Furthermore, the model still benefits from the synthetic simple questions when the scale of labeled data is large.

The main contributions of this paper can be summarized as follows:

- We propose automatically generating large-scale training data for factoid QA via distant supervision. QA of specific domains or new languages, where training data is limited, like medical factoid QA and Vietnamese factoid QA, could benefit a lot from the generated data.
- The generated questions are further adapted to accord with the real-world ones in both content and expression styles by utilizing QA corpus, for example the community QA data. The distant paraphrase approach increases the diversity of query expressions and could improve the generalization of the QA model.
- Experiments are carried out on a real-world QA dataset to validate the proposed approach. Results show that the model can achieve an accuracy of 49.34% without labeled data, and significant improvements are observed when the training data is insufficient. Additionally, the proposal improves the state-of-the-art result on the WebQA dataset [5] from 73.50% to 76.55%. We release our data and codes at [18,19] for reproduction and further research.

The remaining parts of the paper are organized as follows. The related work is reviewed in Section 2. Section 3 introduces the proposed method of generating distant supervised training data, as well as details about the QA model. Experimental results and analysis are given in Section 4. Finally, we conclude the paper and discuss the future work in Section 5.

## 2. Related Work

### 2.1. Document Based Question Answering

Research on document based QA booms from the construction of datasets, such as CNN Daily Mail [10], Squad [4], WebQA [5] and DuReader [20]. Questions from several datasets [4,10] are generated by annotators, while questions from recent datasets [5,20,21] are collected from search engines. For discriminant QA, the answer of the question is a text span of the evidence, while answers for generative QA are generated by annotators. The construction of large-scale question answering datasets enables the training of deep learning models [12,22] with millions of parameters. Typically, a deep learning QA model consists of four layers, namely the embedding layer, the encoding layer, the interaction layer, and the answer layer. The first two layers are also widely adopted in deep learning models for other NLP tasks. In addition the word and character embeddings, syntax features, part of speech features and context vectors [11] are also important for word representation. The attention-based interaction layer is vital for the QA model because it can learn the interaction between the words in the question and the document. Various attention mechanisms are studied in recent work, such as match-LSTM [23], bi-directional attention flow [22], attention-over-attention [24] and self-matching attention [12]. Finally, a pointer network [25] is used to predict the position of the answer span for discriminant QA. In generative QA, answers are generated by a decoder with copy mechanism. Further efforts [26] are also made to study how these QA models answer the questions.

### 2.2. Question Generation for Question Answering

There are also efforts [14,15,27,28,29] focusing on generating questions for QA. Labutov et al. [27] propose an ontology-crowd-relevance method. Firstly, the original document is embedded into a low-dimensional ontology, and then candidate crowd-sourcing templates for question generation are aligned to the ontology, and finally the templates are ranked for a novel region of text. Chali and Hasan [28] aim to generate all possible questions for each topic. Rules are proposed to transform the semantic-role labeled sentences into questions. Song and Zhao [29] generate questions from KBs via templates and then use search engines to perform question expansion, which is similar to our distant paraphrase. However, they do not require the expanded questions to have the same answers with the original questions, so the paraphrased questions could not be used to train a QA model. Du et al. [14] introduce a supervised neural network for question generation. Duan et al. [15] study both retrieval-based and generation-based methods for question generation. They also integrate question generation into an end-to-end QA model, which shows significant improvements. However, their methods are not applicable for the training of QA models with limited annotations because the question generation models are also trained via supervised learning.

To the best of our knowledge, we are the first to generate training data for document-based QA via supervision from KB, and the generated questions are adjusted to reflect the query intentions and expression styles of users.

### 2.3. Distant Supervision

Distant supervision has been studied in a wide range of natural language processing tasks. It is first introduced into the task of relation extraction [30]. Extensive efforts, such as multi-instance learning [31] and selective-instance learning [32,33], have been made to improve the distant assumption so as to reduce the affect of noise. Omer et al. [34] achieve zero shot relation extraction by regarding the relation extraction task as a simple QA task. In addition, distant supervision is also explored in other NLP tasks. Purver et al. [35] test distant supervision on Twitter emotion extraction, and find that the method is suitable for the recognition of happiness, sadness and anger emotions. Plank et al. [36] achieve 8% and 10% error reductions on the Twitter Part of Speech (POS) tagging and Named Entity Recognition (NER), respectively. Without in-domain annotation or hand-crafted rules, Tabassum et al. [37] normalize time expressions in Twitter with a 0.68 F1 score utilizing distant supervision. Zeng et al. [38] generate a large amount of training data for event extraction through distant supervision, improving the detection results of multiple types of events.

Distant supervision is also studied in QA. Several QA datasets [1,39,40] only contain the annotated questions and answers, while the KB entries or texts needed for training are not labeled. Thus, distant supervision is used to determine the evidence KB entries or texts, enabling the supervised learning of QA models [2,40,41,42]. Specifically, Bordes et al. [2] obtain F1 of 62.9% on WebQuestions whose KB entries are distantly annotated. As for answering open-domain questions with Wikipedia, the DrQA model [41] improves 6%, 8.7% and 9.8% on CuratedTREC, WebQuestions, and WikiMovies respectively by utilizing distant supervised annotations of their evidence documents and answer spans. Wang et al. [43] train a model with reinforced learning to rank the texts by the probabilities that correct answers could be generated for given questions, and achieve 2–4 points gains in terms of F1 and EM scores on several datasets. Regarding the candidate documents and document reader as environment, the document ranker learns via explorations and rewards, avoiding the noise caused by the distant supervised labeling of the evidence document. Notice that these works still rely on annotated questions to train the QA model, while we further synthesize the questions utilizing a KB to automatically generate a large-scale training data.

### 2.4. Transfer Learning

Transfer learning, or domain adaptation, aims to transfer knowledge from one domain to another by leveraging the synergistic relationships between different datasets. The source domain is either an unsupervised task or contains large-scale labeled datasets. Transfer learning is widely adopted in deep learning models. Typically, a deep neural network is pre-trained on the source domain, and then fine-tuned on the target domain. For example, the encoder of variational autoencoder [44] or discriminator of generative adversarial networks [45] is used as the feature extractor in semi-supervised learning. Supervised pre-training is commonly performed on ImageNet [46] for computer vision tasks. Typically, only the lowest layer i.e., the word embedding layer, of NLP tasks [12,47,48] is initialized with weights trained in a language model. McCann et al. [11] and Peters [49] respectively utilize the encoders of machine translation and language model to learn context-aware representation of words in multiple NLP tasks including sentiment analysis, question classification, entailment and QA. Transfer learning is also explored in question answering. Several works [50,51] test the transferability between different labeled datasets, and new state-of-the-art results are reported on datasets with limited annotations. Regarding Squad as a large fine-grained labeled answering selection dataset, Sewon et al. [52] achieve significant improvements on WikiQA and SemEval-2016 (Task 3A) via transfer learning. Golub et al. [53] generate synthetic questions and answers using a generative model, and a novel domain-adaptive net is utilized to alleviate the discrepancy between synthetic questions and real-world questions. Their model is trained in a semi-supervised setting, where the generative model and QA model are firstly trained on labeled data and boost each other iteratively. In this paper, synthetic questions and answers are generated utilizing a knowledge base, and the discrepancy between generated data and real-world data is alleviated by exploring community QA.

## 3. Approach

### 3.1. Task Definition

Factoid questions are questions that query concise facts that can be expressed in short texts [54]. In real-world applications, the answer could be generated via a pipeline of a document retriever, an document reader and a optional answer combiner. The document or candidate documents could be retrieved from the document set with a TF-IDF based document retriever like Chen et al. [41] do. Then, a document reader like [12,22,23] extracts answers from the given text. Optionally, an answer combiner [55] merges answers extracted from multiple candidate documents. In this paper, we focus on automatically generating training data for the document reader.

The input of the QA model is a (*q*,*d*) pair, where *q* denotes a factoid question and *d* represents a document. Assuming that the answer is a text span from the given document, then the objective of the QA model is to predict the start position $p_{s}$ and end position $p_{e}$ of the answer span.

In order to train the QA model, we aim to automatically generate a training data set

(1) $$S=\{s_{0},s_{1},\ldots s_{N}\},$$

where $s_{i}$ is a training sample containing the aforementioned four elements for QA model training, specifically

(2) $$s_{i}=\left(q_{i},d_{i},{p_{s}}_{i},{p_{e}}_{i}\right).$$

Additionally, the generated questions should accord with the real-world ones in both query intents and expression styles for generalization ability of the trained QA model.

### 3.2. Training Data Generation via Distant Supervision and Domain Adaptation

In this section, the distant supervision method for automatic training data generation is firstly introduced. After that, details about the domain adaptation on the training data are presented, explaining how to effectively transfer the knowledge of distant-supervised data to answer real-world questions.

#### 3.2.1. Training Data Generation via Distant Supervision

A KB and a corpus are used for the generation of training data. The KB contains triple records *(subject; predicate; object)*, for example *(Albert Einstein; Birth place; Ulm, Kingdom of Württemberg, German Empire)*. Questions are generated by utilizing the template QG(subject,predicate), which takes the subject and predicate as variables. A simple template, “predicate of subject?” can be used, and the synthetic question is “Birth place of Albert Einstein?”. Then, we find the evidence documents and label the answer spans via distant supervision. Documents containing both the subject and object are taken as candidates. The distant supervision assumption is that the candidate document can be used to answer the question and the answer is exactly the mention of the object. For example, the sentence “Albert Einstein was born in Ulm, Kingdom of Württemberg, German Empire, on 14 March 1879” can be taken as evidence, and the answer is labeled as “Ulm, Kingdom of Württemberg, German Empire”. Concentrating on extracting the answer span from the given text, we assume that the given text contains the answer. That is, when generating the data, only texts containing the answer are returned, so we only have positive sentences. The supervision information is the start and end positions of the answer span.

As for data sources for building the corpus, encyclopedias, such as Wikipedia, Baidu Encyclopedia and vertical encyclopedias like IMDB, are good choices. Firstly, these sites are commonly used as the document source for factoid QA because of its coverage, so the language style of the texts is consistent at training and testing. Additionally, the entity-centric characteristic of encyclopedia makes it easier to perform entity linking.

#### 3.2.2. Domain Adaptation

It is obvious that the generated questions are different from the ones asked by users in both the content and the expression style. In order to obtain more natural training data, domain adaptation is performed on the generated questions, by utilizing community QA data that reflects the interests and styles of users.

Firstly, the probability that a synthetic question is asked by users is estimated by searching the QA community with (subject)+(predicate) as a keyword, where the symbol + denotes the logic OR. Specifically, the following factors are considered:

- $N_{Q}$: the total number of retrieved questions.
- $N_{Q_{S}}$: the number of retrieved questions containing the subject.
- $N_{Q_{P}}$: the number of retrieved questions containing the predicate.
- $N_{Q_{O}}$: the number of retrieved questions whose answers contain the object, i.e., the answer of the synthetic question.

Then, the probability of the question being asked is defined as

(3) $$p_{asked}=\alpha_{0}N_{Q}+\alpha_{1}N_{Q_{S}}+\alpha_{2}N_{Q_{P}}+\alpha_{3}N_{Q_{O}},$$

where $\alpha_{*}$ are hyper-parameters. Finally, the probability is normalized, and synthetic questions are selected according to their probabilities, so that the generated training data can possess a more similar distribution with the real-world datasets.

Another problem of the generated data is that the questions’ expressions are monotonous, so the QA model solely trained on these data will be very sensitive to expression variants. Accordingly, a distant paraphrase is proposed to mine paraphrases of questions from the community QA based on distant supervision.

The synonymous relationship between two questions is evaluated from two aspects, namely the contents of questions and their answers. Candidate paraphrases of a synthetic question are firstly retrieved from community QA with (subject)+(predicate) as the keyword. A large portion of questions in the QA forums are discourse questions, but the search procedure can bring a bias/priority towards factoid ones. It is further required that the subject must be mentioned in the candidates. Let $Q^{\prime }$ denote one of the candidate paraphrases, then the assumption for distant paraphrase detection is that $Q^{\prime }$ is accepted if its answer is the object. However, it is a non-trivial task to evaluate whether the answer of a community question is the object because the answer in QA forums usually contains some explanations. Another observation is that answers of factoid questions are usually short and should contain the objects. Thus, for simplicity, the candidate $Q^{\prime }$ is accepted if its short answers contain the object. In the future, we will further study how to identify the equivalent relationship between the object and the answer of a community question. Furthermore, it is also required that $Q^{\prime }$ and the predicate have no words in common, in order to encourage the diverse expressions of predicates.

Supervised learning methods for paraphrase detection of questions are studied in the context of SemEval [56,57]. Aiming at answering new community QA questions, they only use the similarities between questions, while we also consider the similarities between answers. Our architecture may benefit from the combination of these methods in the future.

### 3.3. QA Model

**Figure 1:**
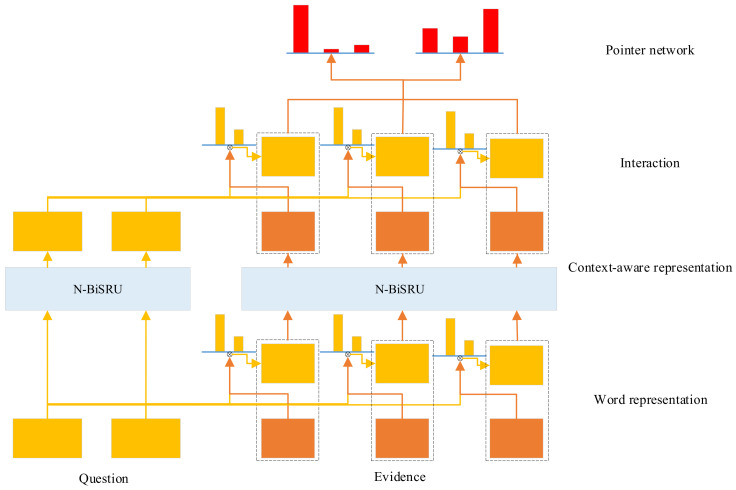
The structure of the QA model.

The QA model adopted in this paper is mainly inspired by DrQA [41] and the attention-based interaction [23] between the question and the document. There are four layers, namely word representation layer, context-aware representation layer, interaction layer and pointer network layer. The word representation layer learns the low-dimensional dense embeddings of words. The context-aware representation layer further enriches the embedding by considering context of the words. The interaction layer learns the interaction between the question and the document via attention mechanisms. Finally, the pointer network layer determines the answer span. The overall structure of the model is illustrated in Figure 1.

#### 3.3.1. Word Representation Layer

Words in the question and the evidence text are modelled on the character level. A modified version of an RNN unit, i.e., simple recurrent unit (SRU) [58], is adopted because it could make better use of the parallel structure of GPU. Given a word *w* that consists of a character sequence

(4) $$C=\{c_{0},c_{1},\cdots ,c_{|w|-1}\},$$

where |w| is the number of characters, a lookup table operation maps every character into the corresponding character embedding

(5) $$C=[c_{0},c_{1},\cdots ,c_{|w|-1}],$$

where $c_{*}\in R^{d_{0}}$ is the $d_{0}$-dimensional character embedding Then, a bidirectional SRU learns the representation of characters, formally

(6) $$H_{c}=Bi-SRU\left(C\right),$$

where $H_{c}\in R^{\left|w\right|\times d_{1}}$ are the hidden states of the SRU, and $d_{1}$ is the size of the hidden state. Finally, max-pooling over time merges the vector sequence into a fixed-size vector as the representation of words

(7) $$w=max\text{-}pool(H_{c}),$$

where $w\in R^{d_{1}}$. Then, the question and the evidence are represented as

(8) $$Q=[q_{0},q_{1},\cdots ,q_{|Q|-1}]$$

and

(9) $$\tilde{D}=\left[{\tilde{d}}_{0},{\tilde{d}}_{1},\cdots ,{\tilde{d}}_{|D|-1}\right],$$

where |Q| and |D| denote the length of the question and the document, respectively.

Furthermore, additional features are considered for the representation of the evidence words. It is observed that the words in question have a lower chance to appear in the answer. Thus, one commonly used binary feature is whether the evidence word also exists in the question. Feature extraction is also performed on the embedding layer. Furthermore, a weighted sum of the words in the question is calculated for every word in the evidence. The weights are calculated by bilinear attention

(10) $$\alpha_{i,j}={\tilde{d}}_{i}^{T}W_{0}q_{j},$$

where $W_{0}\in R^{d_{1}\times d_{1}}$ is a trainable matrix, ${\tilde{d}}_{i}$ and $q_{j}$ denote the representation of *i*-th word of evidence and *j*-th word of question, respectively. Then, the attention weights are normalized by soft-max function

(11) $$a_{i,j}=\frac{exp(\alpha_{i,j})}{\sum_{k=0}^{|Q|-1}\sum_{l=0}^{|R|-1}exp\left(\alpha_{k,l}\right)}.$$

For the *i*-th word of evidence, a weighted sum of the question word representations is calculated as follows:

(12) $${\hat{w}}_{i}=\sum_{j=0}^{|Q|-1}a_{i,j}q_{j},$$

where ${\hat{w}}_{i}$ encodes the most relevant information of the question. Finally, the representation of *i*-th word in the evidence is represented as

(13) $$d_{i}=\left[{\tilde{d}}_{i};{\hat{w}}_{i};f\right],$$

where $d_{i}$ is the embedding of the word, *f* denotes the binary feature, and the symbol ; denotes the concatenation operation, and $d_{i}\in R^{2d_{1}+1}$. Overall, the document is denoted as

(14) $$D=\left[d_{0},d_{1},\cdots ,d_{\left|D\right|}\right]$$

and $D\in R^{\left(2d_{1}+1\right)\times \left(|D|-1\right)}$.

#### 3.3.2. Context Aware Representation Layer

The former layer represents the word with only its characters considered. In this layer, a stacked bi-directional SRU is adopted to learn context-aware representation of the word. Denote a stacked *n* layer bi-directional SRU as n−BiSRU. The question and the document are represented as follows:

(15) $$H=n-BiSRU_{Q}\left(Q\right),$$

(16) $$E=n-BiSRU_{D}\left(D\right),$$

where $H\in R^{2d_{q}\times \left|Q\right|}$, $E\in R^{2d_{d}\times \left|D\right|}$, $d_{*}$ are the sizes of hidden states, respectively.

#### 3.3.3. Interaction Layer

The bilinear attention weights between the document and the question are calculated as follows:

(17) $$B=softmax(E^{T}W_{1}H),$$

where $W_{1}\in R^{2d_{d}\times 2d_{q}}$ is a trainable matrix, and $B\in R^{\left|D|\times |Q\right|}$. Similar to the extraction of an embedding interaction layer, the weighted sum of question word representations is calculated based on the attention

(18) $${\hat{e}}_{i}=\sum_{j=0}^{|Q|-1}B_{i,j}h_{j},$$

where ${\hat{e}}_{i}$ encodes the parts of the question that best match the document word $w_{i}$. Concatenate the representation of *i*-th word $e_{i}$ (*i*-th column of E) in evidence and the weighted sum of question words; then, the interaction space can be denoted as

(19) $$M=\left[\left[e_{0};{\hat{e}}_{0}\right],\left[e_{1};{\hat{e}}_{1}\right],\cdots ,\left[e_{|D|-1};{\hat{e}}_{|D|-1}\right]\right],$$

where $M\in R^{\left(d_{d}+d_{q})\times |D\right|}$, and $m_{i}=\left[e_{i};{\hat{e}}_{i}\right]$ is both context-aware and question aware.

#### 3.3.4. Pointer Network Layer

The span of the answer is predicted by the pointer network layer. Two classifiers are adopted to predict the probability of start and end positions, formally

(20) $$S_{i}=\sigma \left(w_{2}m_{i}\right),$$

(21) $$E_{i}=\sigma \left(w_{3}m_{i}\right),$$

where σ is the logistic activation, $m_{i}$ is the *i*-th column of matrix M, and $w_{2},w_{3}\in R^{2d_{d}+2d_{q}}$ are trainable parameters. Then, $S_{i}$ and $E_{i}$ are the probability of the *i*-th word in the evidence being the start and end of the answer span. Finally, constrained inference is performed to determine the answer span. Let the score of an span denoted by

(22) $$s_{ij}=S_{i}+E_{j},$$

then the span (i,j) with the highest score and satisfying the constraint i≤j≤i+L is taken as the answer span, where *L* is the length of the longest span.

### 3.4. Training

The QA model is supervised by utilizing the cross entropy loss at the start and end position. Formally, we have the loss of the QA model defined as follows:

(23) $$L=-\frac{1}{N}\sum_{k=0}^{N-1}\left[log\left(S_{s_{k}^{g}}\right)+log\left(E_{e_{k}^{g}}\right)\right],$$

where $s_{k}^{g}$ and $e_{k}^{g}$ are the golden start and end positions of the *i*-th sample, respectively, and *N* is the size of batch.

Backward propagation is adopted to update the model parameters

(24) $$\theta =\theta -\lambda \frac{\partial L}{\partial \theta },$$

where θ is the parameters in the QA model, and λ is the learning rate. The Adamax algorithm [59] is used to adjust the learning rate.

## 4. Experiments

### 4.1. Dataset

WebQA [5] is a large-scale factoid QA dataset. The questions in the dataset are real-world ones mined from the Baidu search engine (https://www.baidu.com/), containing both simple and complex questions. More details about the construction of WebQA can be found in [5]. There are 140,897, 3018, and 3024 question answering pairs in the training, validation, and test dataset, respectively.

Since WebQA is a Chinese factoid dataset, a Chinese KB is needed. Considering that predicates in DBpedia are in English, we construct a new Chinese KB leveraging Baidu Baike (https://baike.baidu.com), the largest Chinese encyclopedias. More than 1.5 million items are crawled from Baidu Baike during September 2017. Finally, 3.15 million triples are extracted from the info-boxes of entity pages to form the KB, and the corpus is constructed by extracting the abstracts of the pages. The community QA site, Baidu Zhidao (https://zhidao.baidu.com), is explored for distant QA pairs selection and distant paraphrase.

Utilizing the KB and corpus, 1.05 million training QA pairs are generated by the distant supervision method introduced in Section 3. The hyper-parameters for evaluating $p_{asked}$ are set to 0.1, 0.4, 0.6, 0.8 for $\alpha_{0},\alpha_{1},\alpha_{2},\alpha_{3}$, respectively. In addition, 5767 paraphrased questions with diverse expressions are mined for 2552 triples using the proposed distant paraphrase method. There is a trade-off between the precision and recall, so a large number of correct paraphrased questions are dropped to avoid the noise in mined results. We would design a model to reduce the impact of noise to recall more distant paraphrased questions in the future. We also release this data for further research on paraphrase mining.

**Figure 2:**
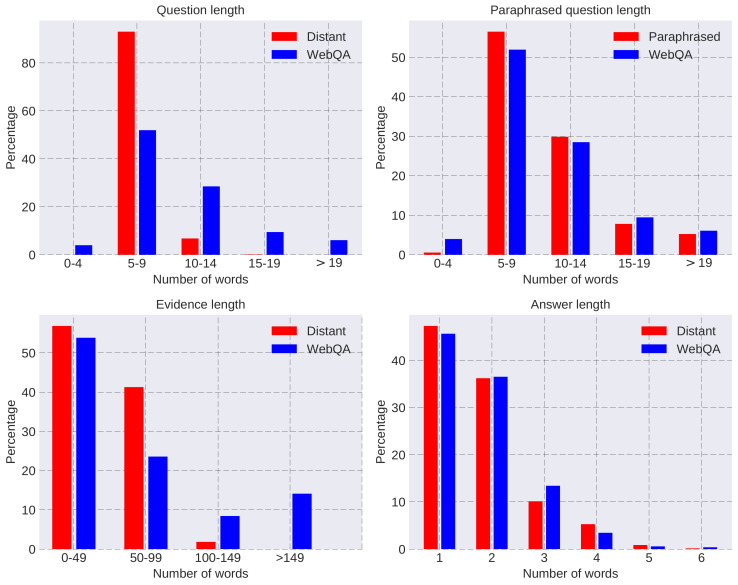
Statistics of WebQA and training data generated via distant supervision.

**Figure 3:**
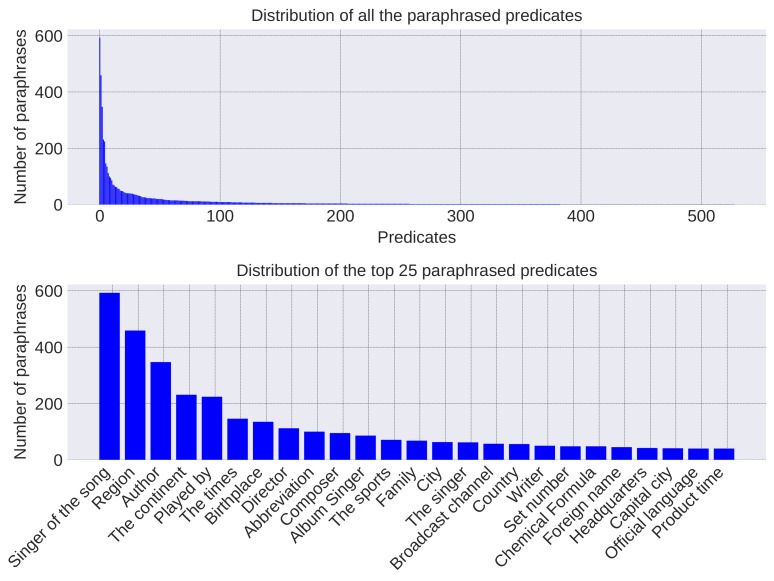
Distribution of the paraphrased predicates.

Statistics of the length of the generated questions, evidence, and answers are given in Figure 2. The distribution of the answers’ length is similar to that of WebQA. There are few long pieces of evidence in generated data because the evidence is partitioned into sentences during preprocessing. As for questions, the sentence structures of synthetic questions are monotonous since they are generated via the simple template. Most of the synthetic questions have 5–9 words because the entity name and predicate contain 1–5 and 1–2 words, respectively. By contrast, the length of paraphrased questions has a similar distribution with WebQA, and these questions have diverse and flexible expressions since they are mined from Community QA data. Statistics of the paraphrased predicates are given in Figure 3. The distribution of paraphrases is skewed and paraphrases of the top 100 predicates make up a large proportion. The top 25 predicates are listed in the second sub-plot. It can be seen that diverse topics such as music, sports and geography are included.

To evaluate the quality of the distant paraphrased questions, 100 of them are randomly sampled and manually labeled. The statistic result and samples of the distant paraphrases are given in Table 2. In addition, 61 of the paraphrases are correct, and the errors are roughly categorized into four overlapped categories/causes, namely multiple query intents (MQI), not exactly matching (NEM), failure of supervision (FoS) and discourse questions (DQ). MQI errors are caused by the phenomenon that users of QA forums usually have multiple related query intents in a single question. NEM means the same aspect of entity is queried, but the expected answers are from different granularities. For example, the questions “which province is Xi’an in?” and “region of Xi’an?” both query the location of Xi’an. However, the expected answers are from different levels. We assume that retrieved questions are paraphrases of the synthetic questions when the object is contained in the answer. FoS happens when the object is mentioned in the answer, but other relations are described. A large portion of questions in the community QA forums are discourse questions on opinionated, experiential or controversial topics, and a selection of these questions causes the DQ errors.

### 4.2. Experiment Settings

**Figure 4:**
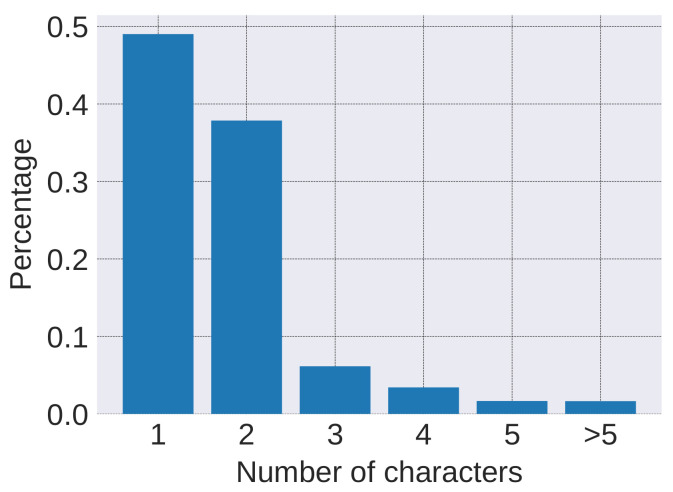
Word length distribution.

The main hyper-parameters of the model are summarized in Table 3. The dimensions of character embedding and word embedding are both set to 64 following the work [5]. The number of characters in a word is determined according to the statistics of the length of words. As is illustrated in Figure 4, most (90%+) of the Chinese words are within three characters. Thus, the max-length of characters is set to 3 for a trade-off between computation complexity and accuracy. In addition, the number of training epoch is determined by the results on the validation set.

We use the validation and test set of WebQA to evaluate the result. The performance of the model is measured by accuracy

(25) $$A=\frac{\left|C\right|}{\left|Q\right|},$$

where *Q* is the list of questions, and *C* is the questions that are answered correctly. There are two ways, namely strict and fuzzy manners introduced by Li et al. [5], to evaluate whether a question is answered correctly. In the strict manner, a question is considered as correctly answered when the generated answer exactly matches the labeled answer. In fuzzy manner, the answer is considered as correct if the generated answer is a synonym of the golden answer. For example, “Beijing City” is a synonym of “Beijing”. In this paper, we use the fuzzy match accuracy $A_{FM}$ as the criterion. Let $|Q_{r}|$ denote the number of answers returned by the system, the precision and recall are calculated as

(26) $$P=\frac{\left|C\right|}{|Q_{r}|},$$

(27) $$R=\frac{\left|C\right|}{\left|Q\right|}.$$

Since the pointer network generates one answer for each question, our system will return |Q| answers, i.e., $|Q_{r}\left|=|Q\right|$. As a result, precision, recall and F1 score are equal to the accuracy for our method.

### 4.3. Experimental Results and Analysis

#### 4.3.1. Factoid QA with Only Distantly Supervised Training Data

In this subsection, we introduce the results obtained by utilizing the distantly supervised data. Let $N_{dis}$ denote the number of distantly labeled QA pairs for training the model, and three configurations are set as follows:

- DSBasic (Distant Supervision Basic): all the training samples generated by distant supervision are equally treated, and $N_{dis}$ QA pairs are randomly selected.
- DS+SS (Distant supervision with sample selection): the generated samples are weighted by the probability $p_{asked}$, and QA pairs with the top $N_{dis}$ $p_{asked}$ are selected. In the experiments, synthetic questions with probability 0 are included when $N_{dis}$ is larger than the number of generated questions with non-zero probability.
- DS+SS+DP (DS+SS with disant paraphrase): questions with the top $N_{dis}-N_{DP}$ $p_{asked}$ are selected, where $N_{DP}=5767$ is the number of paraphrased questions. Then, $N_{DP}$ QA pairs with paraphrased questions are added.

**Figure 5:**
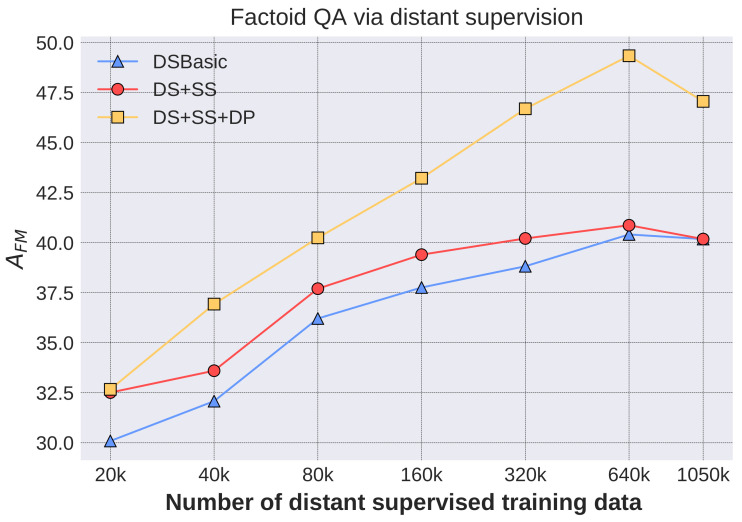
Factoid QA via distant supervision.

Factoid QA results under the above three configurations are given in Figure 5. Our QA system achieves an fuzzy matching accuracy of 49.34% with only training data obtained via distant supervision, indicating that the proposed method is promising. Some other interesting facts can also be observed from the results. Firstly, the QA performance improves with the increase of distant-supervised training samples. Secondly, the DS+SS method outperforms DSBasic by two points, benefiting from the domain adaptation operation by selecting important samples according to probabilities derived from community QA data. In addition, sample selection can also improve the model’s efficiency when the computing capabilities are limited while the scale of distant-supervised data is huge. The effectiveness of sample selection decreases at $N_{dis}=640$ k and $N_{dis}=1050$ k because there are only 1050 k candidate samples and most of them are kept. Thirdly, the more distantly supervised samples are used, the larger improvement is brought by the distant paraphrase. The model solely trained on large scale distantly supervised samples tends to be a good information extractor but not good at understanding diverse query expressions. Adding the distant paraphrased samples alleviates the bottleneck. For example, about six and nine point improvements (when the improvement is of six points, $\chi^{2}$ = 25.85, $\chi_{p=0.001}^{2}$ = 10.83, so *p* < 0.001) are obtained with training size 320 k and 640 k, respectively.

We further analyse the influence of distant paraphrase on the model’s generalization ability to answer real-world questions through case studies. Answers of three questions with the same query intent are given in Table 4. The model trained on synthetic data could answer the first question because the question has a similar schema with the synthetic questions, but the other two questions are mistakenly answered. Apparently, the model is sensitive to the variants of expressions though the changes are small for humans. This is because synthetic questions are all simple, so the model has little chance to see other auxiliary words and learn from them. By contrast, the model trained with distant paraphrases is robust to the W-words, such as where, when and who, and can correctly answer three questions, showing its good generalization ability.

#### 4.3.2. Improved Factoid QA with Distant Supervision

In this subsection, we evaluate whether factoid QA could benefit from the synthetic training data generated by distant supervision when some labeled data is available. Different portions of the 140 k QA pairs (WebQA training set) are kept to simulate different amounts of available labeled data. Experimental configurations are as follows:

- SLBasic (Supervised Learning Basic): as the baseline, the QA model is trained solely on the labeled data.
- DS+SL: the QA model is pre-trained on 320 k QA pairs generated via distant supervision (DSBasic) and then trained on the labeled data of the same size as SLBasic.
- DS+SS+SL: the QA model is pre-trained on 320 k QA pairs generated via distant supervision (DS+SS) and then trained on the labeled data of the same size as SLBasic.
- DS+SS+DP+SL: the QA model is pre-trained on 320 k QA pairs generated via distant supervision (DS+SS+DP) and then trained on the labeled data of the same size as SLBasic.

**Figure 6:**
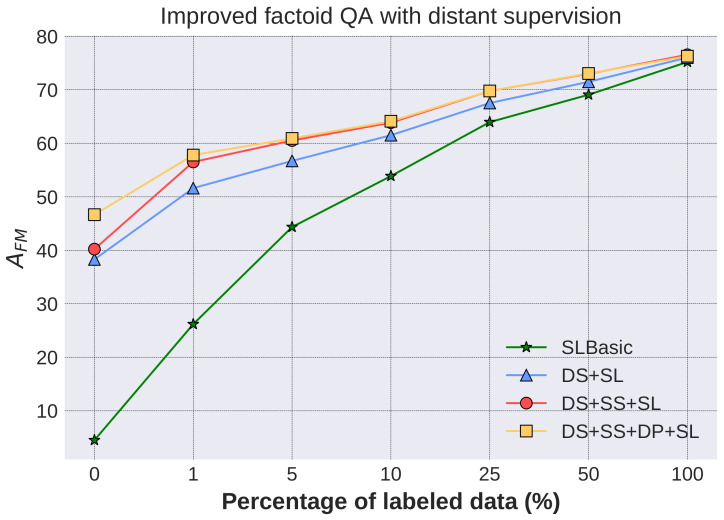
Improved factoid QA with distant supervision.

Performances of the models under different amounts of labeled data are illustrated in Figure 6. Using the same amount of labelled data, models pre-trained on distantly supervised data outperform SLBasic, proving that our method could be used to improve the QA performance of domains where the amount of labeled data is limited. For example, with approximately 14 k (10%) labeled data, the results of the models with and without pre-training are 63.86% (DS+SS+SL) and 53.87% (SLBasic) respectively. In particular, the results are significantly improved when there is a small amount of training data. For example, when 1% (approximately 1.4 k) of the labeled data is given, the model using distant supervision data generated via DS+SS improves $A_{FM}$ from 26.19% to 56.51%. Meanwhile, the model can still benefit from learning to answer the simple synthetic questions when there is lots of training data. Specifically, with 100% of the labeled samples, our method gets an absolute increase of 1.35 $A_{FM}$ points over the baseline model trained on the WebQA data. The DS+SS+SL outperforms DS+SL by 2–5 points, demonstrating that the synthetic question selection is effective. DS+SS+DP+SL only outperforms DS+SS+SL when training data is very limited (0–1.4 k) because the bottleneck of understanding diverse query expressions could also be alleviated by the annotated data. The scale of paraphrases is small compared to the labeled set and there is noise in the mined paraphrases. Nevertheless, a distant paraphrase could potentially assist annotation of QA pairs.

We further analyse how the QA models are enhanced using different approaches to incorporate the generated data. The adopted configurations are as follows:

- Supervised learning (SL): The model is solely trained on annotated QA pairs.
- Pre-training+ SL: The model is pre-trained on 320 k generated QA pairs (generated via DS+SS configuration) and then trained on the annotated data.
- SL+: The model is simultaneously trained on both the generated data and annotated data. Specifically, the model is iteratively trained on a mini-batch of generated data and another mini-batch of annotated data. Note that the training loss and mini-batch number are calculated and counted on the annotated data.

Experimental results of different labeling rate (the percentages of labeled data used to train the models) are listed in Table 5. It is observed that incorporating generated data to the labeled data improves the model’s performance, both in the pre-training + SL and SL+ configurations.

**Figure 7:**
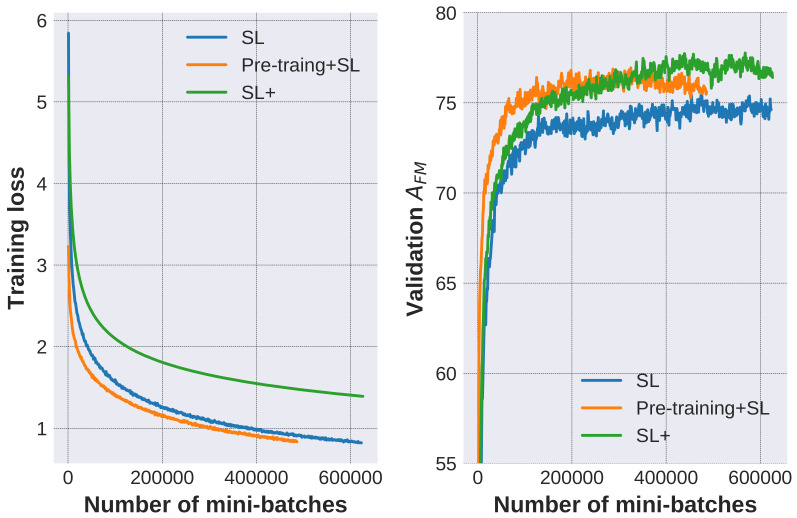
Curves of training loss and validation accuracy. SL denotes supervised learning. Pre-training+ SL denotes that the model is pre-trained on generated data and then trained on the annotated data. SL+ denotes the model simultaneously trained on generated data and annotated data.

The curves of training loss and validation accuracy under 100% labeling rate are given in Figure 7, where the fuzzy accuracy is reported every 1000 mini-batches and the losses are the average of every 1000 mini-batches. It is observed that the training loss of Pre-training+SL decreases faster than SL, and the fuzzy accuracy improves faster. This observation indicates that the model pre-trained on the generated data could extract relevant features and obtain better generalization ability. This is similar to the effect of the classical pre-training of DNN classifier using an auto-encoder [60]. Answering the synthetic questions and reconstructing the figures could both be regarded as auxiliary tasks. The main difference is that our auxiliary task could use the identical neural networks with the main task and requires a similar abstraction level of features. Meanwhile, compared with SL, SL+ has a higher training loss and achieves higher validation accuracy. This is because both SL and SL+ have the same parameter size, while the latter learns to answer both annotated questions and synthetic ones, so its Rademacher complexity, or the ability to fit random noise, is reduced to decrease the risk of over-fitting.

We further compare the proposed method with the sequence-labeling methods [5]. More experiments are also carried out with two state-of-the-art QA models, i.e., the Bi-Directional Attention Flow model (BiDAF) [22] and R-NET [12], in order to investigate the generalization of our distant supervised learning method. Details of the models are as follows:

- Sequence-labeling methods [5]: the question is first encoded into a vector utilizing single-time attention. Then, question-aware representations of evidence words are learned with bi-directional LSTMs. Finally, a softmax or CRF layer is used to predict the labels. The sequence-labeling methods are capable of generating zero, one or multiple answers for a question and a given document, thus precision (P), recall (R) and F1 scores are used in the evaluation.
- Methods with interaction attention and pointer net: BiDAF [22], R-NET [12] and our baseline all adopt interaction attention and pointer net. These models all contain word–word interactions between the question and the evidence, which are supposed to better perform question aware reading comprehension [23]. BiDAF contains both context to query and query to context attention. Self-attention and several gates are adopted in R-NET considering that only parts of the document contribute to the answer extraction. Our baseline method contains question to document interaction in two layers. Character-level encoding and the binary feature *f* are used in word representation of these three models.
- Methods with interaction attention and pointer net + DS: models are the same as those of the previous configuration. The only difference is that 320 k generated QA pairs (under DS+SS configuration) are added to the annotated data.

Experimental results are listed in Table 6. Sequence-labeling methods could generate multiple answers from a QA pair, so a sequence-labeling model with CRF obtains the highest recall. In the meantime, the precision of these systems decreases. When comparing with the results, one should notice their flexibility and capability to generate zero, one or multiple answers. BiDAF, R-NET and our baseline outperform sequence-labeling methods on F1 value, showing the importance of interaction attention, and our baseline model achieves competitive results with BiDAF and R-NET. When optimized on both generated data and labeled data, BiDAF, R-NET and our baseline achieve the state-of-the-art results, and improvements of roughly one point are observed. With 50% of the annotated data, incorporating distant supervision could bring more significant improvements to the three models, adding 2.25, 2.22 and 3.90 points for BiDAF, R-NET and our baseline model, respectively. Generally, the distant supervision data generation method could boost the performance of QA models, demonstrating its potential in QA of new domains or languages where annotated data is limited.

## 5. Conclusions

Motivated by the fact that it is very expensive to label data for deep learning QA models, we explore the method of generating large-scale training data for factoid QA via distant supervision. Additional efforts are made to adapt the generated data to accord with real-world ones. Specifically, we select the generated questions that can better reflect the query interests of users. Paraphrases of questions are mined from community QA data to enrich the expression styles of the generated data. Experimental results on a real-world QA dataset show that the deep learning QA model benefits from learning to answer the generated questions, even when a large-scale training data is available, demonstrating the potential of our proposal in factoid QA of new domains or languages. To the best of our knowledge, this paper makes the first attempt to answer real-world questions by utilizing distant supervision.

In future work, iterative paraphrase mining and QA model training will be studied. On one hand, the QA model, which could be used to extract the exact answer of a community question, is helpful for distant paraphrase. On the other hand, the QA model benefits from the mined question paraphrases that have less noise. In addition to the QA model, the document retriever is also an important component of a document based QA system. In this paper, we only generate training data for the QA model and test the model in an environment where the correct document is provided. In the future, we will incorporate the document retriever, exploring methods that can reduce the side effect of the mistakenly retrieved documents and better utilize the multiple retrieved documents to verify the answer. In addition, we will also investigate how to generate training data for the document retriever using distant supervision, in order to build a holistic QA system that can be generalized to new domains or languages. 

## Figures and Tables

**Table 1:** Illustration of distantly supervised training data generation for Factoid QA.

**Triple**	Subject	Albert Einstein
	Predict	Birth place
	Object	Ulm, Kingdom of Württemberg, German Empire
**Question**	Birth place of Albert Einstein?	
**Answer**	Ulm, Kingdom of Württemberg, German Empire	
**Evidence**	Albert Einstein was born in Ulm, the Kingdom of Württemberg in the German Empire, on 14 March 1879.	

**Table 2:** Statistics and samples of paraphrased questions, where MQI, FoS, NEM and DQ denote Multiple Query Intents, Failure of Supervision, Not Exactly Matching and Discourse Questions respectively.

Tag	Num.	Synthetic Question	Mined Paraphrase of the Question	Error Cate.
Correct	61	Author of Pride and Prejudice?	Who wrote the book Pride and Prejudice?	-
		Spouse of Barack Obama?	Who is Barack Obama’s wife?	
Error	39	Nation of Odyssey?	Which ancient country did Odyssey and Ilias belong to?	MQI
		Athletics items of YangWei?	What sport did YangWei and Li Xiaopeng play?	MQI
		Composer of Sambo auspicious?	Who writes the lyrics and composes for Sambo auspicious?	MQI, FoS
		Region of Xi’an?	Which province is Xi’an in?	NEM, FoS
		Author of Pride and Prejudice?	Briefly introduce the writer of Pride and Prejudice.	NEM, DQ
		Producer of Black Humor?	Who composes for Black Humor?	FoS
		Country of Three Meals a Day?	On which website could I watch Three Meals a Day?	FoS
		Abbreviation of LuXian No.2 High School?	LuXian No.2 and LuZhou No.2, which is better?	DQ
		Original singer of DjKunsonRMX?	Who sings DjKunsonRMX better, Guanjie Xu or Baiqiang Chen?	DQ

**Table 3:** Hyper-parameters of the QA model.

Parameter	Parameter Value
Dim. of character embedding $d_{0}$	64
Dim. of word embedding $d_{1}$	64
Num. of characters in a word	3
Dim. of hidden SRU layer $d_{q},d_{d}$	100
Num. of stacked SRU layers	9
Dropout rate of embedding	0.5
Dropout rate of SRU output	0.15
Learning rate λ	0.001

**Table 4:** Case study for the influence of distant paraphrase. The three questions are of the same query intent but different expressions. It is observed that the model trained with some distant paraphrases is more robust. DS, SS and DP are short for Distant Supervision, Sample Selection and Distant Paraphrase respectively.

Method	Question	Evidence	Answer Generated	Score
DS+SS	Born place of Archimedes?	…	Sicily	0.71
	Where is the born place of Archimedes?	In 287 BC, Archimedes was born in Sicily (now Italy Siracusa)	Archimedes was born in Sicily	0.36
	Where was Archimedes born?		Archimedes	0.59
DS+SS+DP	Born place of Archimedes?		Sicily	0.59
	Where is the born place of Archimedes?		Sicily	0.50
	Where was Archimedes born?	…	Sicily	0.46

**Table 5:** QA performances using different approaches to incorporate the generated data. Here SL is short for Supervised Learning.

Configuration	Labeling Rate (%)	$A_{\mathrm{fm}}$
SL	50	69.08
Pre-training + SL	50	72.98
SL+	50	72.02
SL	100	75.20
Pre-training + SL	100	76.55
SL+	100	77.25

**Table 6:** Performances with different methods and labeling rates. Here DS is short for distant supervision.

Model Class	Method	Labeling Rate (%)	P (%)	R (%)	F1 (%)
Sequence-labeling	Seq-labeling with Softmax [5]	100	63.58	73.63	68.24
	Seq-labeling with CRF [5]	100	67.53	80.63	73.50
Interaction attention and pointer net	BiDAF [22]	100	74.54	74.54	74.54
	R-NET [12]	100	75.36	75.36	75.36
	Our baseline	100	75.20	75.20	75.20
Interaction attention and pointer net + DS	BiDAF [22] + DS	100	75.66	75.66	75.66
	R-NET [12] + DS	100	76.22	76.22	76.22
	Our baseline + DS	100	76.55	76.55	76.55
Interaction attention and pointer net	BiDAF [22]	50	70.27	70.27	70.27
	R-NET [12]	50	70.23	70.23	70.23
	Our baseline	50	69.08	69.08	69.08
Interaction attention and pointer net + DS	BiDAF [22] + DS	50	72.52	72.52	72.52
	R-NET [12] + DS	50	72.45	72.45	72.45
	Our baseline + DS	50	72.98	72.98	72.98

## Abbreviations

The following abbreviations are used in this manuscript:

QA	Question Answering
KB	Knowledge Base
NLP	Natural Language Processing
LSTM	Long Short-Term Memory
TF-IDF	Term Frequency–Inverse Document Frequency
RNN	Recurrent Neural Network
SRU	Simple Recurrent Unit
CRF	Conditional Random Field
BiDAF	Bi-Directional Attention Flow
DS	Distant Supervision
